# Supplementary material for: The Coronavirus Pandemic and the Occurrence of Psychosomatic Symptoms: Are They Related?
Source: Int J Environ Res Public Health. 2021 Mar 30;18(7):3570. doi: 10.3390/ijerph18073570 (PMC8036614; doi:10.3390/ijerph18073570)
Supplement: Supplementary file 1 [file ijerph-18-03570-s001.pdf]

Supplementary Material

Table S1. Associations of increased frequencies of health complaints with sociodemographic factors – crude (odds ratios – OR, and 95% confidence intervals – 95% CI).

	**Headache**	**Stomach Ache**	**Backache**	**Intestinal Problems**	**Feeling Low**	**Irritability**	**Nervousness**	**Sleeping Difficulties**	**Dizziness**
Age	0.98 (0.97–0.99) ***	0.99 (0.94–1.00) *	1.00 (0.99–1.01)	0.99 (0.98–1.00)	0.99 (0.98–1.00) **	0.99 (0.98–1.00) **	0.99 (0.98–1.00) **	0.99 (0.98–1.00) **	0.98 (0.96–1.00) *
Gender									
Male	1	1	1	1	1	1	1	1	1
Female	1,26 (0.97–1.64)	1.86 (1.26–2.74) **	1.12 (0.82–1.53)	1.11 (0.75–1.65)	2.27 (1.71–3.01) ***	1.33 (1.01–1.74)*	1.53 (1.17–2.00) **	1.25 (0.90–1.73)	1.86 (1.03–3.34) *
Highest achieved education									
Elementary school	1	1	1	1	1	1	1	1	1
Secondary vocation school	1.58 (0.89–2.81)	0.47 (0.26–0.84) *	1.00 (0.56–1.77)	0.56 (0.29–1.09)	0.69 (0.42–1.15)	1.03 (0.61–1.76)	0.76 (0.46–1.24)	0.66 (0.38–1.16)	0.38 (0.16–0.91) *
Secondary school with graduation	2.00 (1,12–3,58) *	0.47 (0.26–0.87) *	0.85 (0.47–1.54)	0.81 (0.42–1.57)	1.11 (0.67–1.83)	1.29 (0.75–2.21)	1.05 (0.64–1.73)	0.70 (0.40–1.27)	0.56 (0.24–1.32)
College	1.57 (0.81–3.03)	0.59 (0.29–1.20)	1.20 (0.62–2.32)	0.59 (0.26–1.34)	1.49 (0.85–2.61)	1.77 (0.98–3.20)	1.37 (0.89–2.39)	0.91 (0.48–1.76)	0.57 (0.20–1.61)
Economic status									
Household/unemployed	1	1	1	1	1	1	1	1	1

Student	1.87 (0.98–3.55)	0.85 (0.37–1.93)	1.28 (0.57–2.86)	1.49 (0.65–3.42)	1.55 (0.83–2.92)	2.02 (1.02–3.99) *	1.63 (0.86–3.06)	2.43 (1.17–5.06) *	2.06 (0.61–7.00)
Disabled/Old–aged pensioner	0.65 (0.39–1.07)	0.44 (0.24–0.80) **	1.07 (0.59–1.94)	0.58 (0.30–1.13)	0.59 (0.36–0.95) *	0.95 (0.56–1.62)1	0.76 (0.47–1.22)	0.83 (0.46–1.52)	0.90 (0.33–2.52)
Employee	0.97 (0.60–1.55)	0.43 (0.25–0.76) **	0.94 (0.53–1.66)	0.56 (0.30–1.06)	0.64 (0.41–1.01)	1.20 (0.73–1.99)	0.70 (0.44–1.10)	0.77 (0.43–1.38)	0.71 (0.26–1.93)
Self–employed	0.94 (0.45–1.94)	0.81 (0.35–1.90)	1.25 (0.55–2.87)	0.73 (0.27–2.00)	0.92 (0.46–1.84)	0.72 (0.31–1.67)	0.68 (0.32–1.42)	0.75 (0.29–1.91)	1.06 (0.25–4.58)
Marital status									
Single/Divorced/Widow(er)	1	1	1	1	1	1	1	1	1
Married/Partner relationship	1.14 (0.86–1.51)	0.96 (0.65–1.42)	0.99 (0.71–1.37)	0.80 (0.54–1.20)	0.86 (0.65–1.14)	0.82 (0.62–1.09)	0.82 (0.62–1.08)	1.08 (0.76–1.53)	1.00 (0.56–1.83)
Faith									
Non–religious	1	1	1	1	1	1	1	1	1
Religious	1.24 (0.94–1.63)	1.15 (0.78–1.69)	0.95 (0.68–1.32)	1.07 (0.71–1.61)	1.18 (0.89–1.57)	0.93 (0.70–1.23)	1.20 (0.91–1.58)	1.17 (0.84–1.65)	1.56 (0.88–2.76)
Spirituality	1.00 (0.99–1.01)	1.01 (1.00–1.02) *	1.00 (0.99–1.01)	1.01 (0.99–1.02)	1.01 (1.00–1.02) *	1.00 (0.99–1.01)	1.01 (1.00–1.02)	1.00 (0.99–1.01)	1.03 (1.01–1.04) **

Notes: * $p < 0.05$, ** $p < 0.01$, *** $p < 0.001$; after using Bonferroni correction, only p–values below 0.01 are considered significant.
